# Why HALO 301 Failed and Implications for Treatment of Pancreatic Cancer

**DOI:** 10.17140/POJ-3-e010

**Published:** 2019-12-20

**Authors:** Nausheen Hakim, Rajvi Patel, Craig Devoe, Muhammad W. Saif

**Affiliations:** Northwell Health Cancer Institute and Donald and Barbara Zucker School of Medicine, Hofstra/Northwell, NY, USA

**Keywords:** Pegvorhyaluronidase alfa (PEGPH20), Desmoplasia, Pancreatic cancer, Chemoresistance, HALO

## Abstract

Survival rates for pancreatic cancer (PC) remain dismal. Current standard of care treatment regimens provide transient clinical benefit but eventually chemoresistance develops leading to poor outcomes. PC is a relatively chemoresistant tumor and one of the explanations for this is attributed to desmoplasia that impedes drug delivery. Based on this, stromal modifying agent such as Pegvorhyaluronidase alfa (PEGPH20) was developed and investigated in phase I-III studies. Although phase I-II studies showed promising results in patients with high hyaluronic acid (HA) expressing tumors, the phase III HALO 301 study failed to miss it’s primary endpoint and further development of PEHPH20 is halted. This failure implies that targeting desmoplasia alone is not sufficient and other intrinsic factors such as lack of significant neoantigens, low tumor mutational burden, and epithelial to mesenchymal transition may be at play. It is also important to consider that although the tumor stroma may be a physical barrier hampering drug delivery, it may also have protective effects in restraining tumor growth and progression. Further studies in molecular biology to better characterize the complex interaction between the microenvironment and cancer cells are warranted.

Pancreatic cancer (PC) remains one of the deadliest cancers in the United States with very poor outcomes. In 2019, it is estimated that 56,770 Americans will be diagnosed with pancreatic cancer in the US and more than 45,750 will die of the disease. It is expected to become the second leading cause of cancer-related deaths by 2020 and currently has a 5-year overall survival rate of 8.5% for all stages combined.^1^ PC is a relatively chemoresistant tumor with limited treatment options, which can include surgery if identified early in the disease process, radiation, chemotherapy, and some targeted therapies. Most patients present with unresectable or metastatic disease resulting in a 5-year-overall survival (OS) rate of only 7%. Even when surgery is feasible in 15–20% of the patients, the 5-year survival remains only about 10%.^2^

Chemotherapy, which can be used in the neoadjuvant setting in order to decrease tumor size in the borderline resectable or resectable patients, in the adjuvant setting after surgery, or first line in the metastatic/advanced setting, is the forefront of systemic therapy. Two chemotherapy combination regimens have shown superiority in patients with metastatic disease. In the Partenariat de Recherche en Oncologie Digestive (PRODIGE)/Actions Con-certées dans les Cancers Colorectaux et Digestifs (ACCORD) and Metastatic Pancreatic Adenocarcinoma Clinical Trial (MPACT) trials, folinic acid fluorouracil irinotecan oxaliplatin (FOLFIRINOX) and gemcitabine plus nab-paclitaxel, respectively, showed an overall survival (OS) benefit at the cost of increased toxicity.^3,4^ In 2007, the Food and Drug Administration (FDA) approved erlotinib (epidermal growth factor receptor tyrosine kinase inhibitor) based on a study that showed survival benefit with combination of gemcitabine plus erlotinib, however, clinical benefit is limited and erlotinib is not widely accepted or used.^5^ A recent study using olaparib (poly (adenosine diphosphate [ADP]–ribose) polymerase-PARP inhibitor) in the maintenance setting in patients with germline *BRCA* mutated metastatic pancreatic cancer that had not progressed on first line platinum based therapy showed significant improvement in median progression free survival (PFS) but no improvement in OS.^6^ However, this only applies to a small subgroup of patients with pancreatic cancer. Despite these advances, there remains much room for improvement and one of the explanations for resistance to conventional chemotherapy can be attributed to desmoplasia that impedes drug delivery.

Pancreatic tumor cells have a thick and poorly perfused stroma, which includes pancreatic stellate cells (PSCs). These cells, in turn, produce stromal elements, including collagens, laminin, fibronectin, and hyaluronic acid (HA). The dense, collagen-rich extracellular matrix and stroma creates high interstitial pressure. This can potentially constrict blood vessels leading to a hypovascular environment that impairs drug delivery rendering tumor cells chemoresistant.^7^ This fundamental characteristic of significant over-production of extracellular matrix proteins and extensive proliferation of myofibroblast-like cells is termed desmoplastic reaction and can decrease the efficacy of chemotherapy.

Tumors with high-levels of HA have shown to be poor prognostic indicators in patients with PC.^8^ Stromal modifying agents, such as Pegvorhyaluronidase alfa (PEGPH20), an enzyme that temporarily degrades HA, should decrease tumor pressure and vascular compression thereby penetrating the “halo” surrounding the tumor cells. PEGPH20 showed promising results in preclinical and early phase studies. Based on this the FDA granted orphan drug designation to PEGPH20 for treatment of PC.

Encouraged by the preliminary data from phase I/II studies, the phase III HALO-109–301 was conducted which was a randomized, double-blind, multicenter trial.^9,10^ This study compared PEGPH20 in combination with nab-paclitaxel/gemcitabine to nab-paclitaxel/gemcitabine alone in previously untreated patients with stage IV PC.^11^ Patients had to have tumors that were high expressors of HA, a clear difference from SWOG1313. Patients were randomized in 2:1 manner to receive the experimental arm. The primary endpoint was overall survival, and the originally planned interim analysis was not done. Five hundred patients were enrolled and estimated completion date was to be December of 2019. However, on November 4, 2019, Halozyme announced that HALO 301 did not meet its primary endpoint of OS (11.2 months compared to 11.5 months (hazard ratio of 1.00 and *p*=0.096).^12^ Per Halozyme, there was a higher response rate in the experimental arm but unfortunately no improvement in duration of response, PFS, or OS was seen. Since this announcement, all future development of PEGPH20 has been halted.

Earlier in 2019, Ramanathan et al, presented similar negative data of Southwest Oncology Group (SWOG) 1313 that was conducted to determine the safety and efficacy of PEGPH20 in combination with modified version of FOLFIRINOX. Since 2011, when an exceptional improvement in overall survival with FOLFIRINOX compared with single agent gemcitabine (HR for death=0.57) was published,^3^ a substantial interest had developed to use this new triplet chemotherapy as the backbone for a randomized clinical trial with PEGPH20. The SWOG set out to determine the safety and efficacy of PEGPH20 in combination with modified version of FOLFIRINOX in a phase Ib/II clinical trial.^13^ Of note, eligibility of subjects was not dependent upon tumor expression status of HA by immunohistochemistry. The standard 3+3 dose escalation phase Ib component demonstrated that biweekly dosing of PEGPH20 was poorly tolerated, and the RP2D was 3 μg/kg every 2 weeks. The open-label, phase 2 portion randomly assigned 114 patients to mFOLFIRINOX+/−PEGPH20. However, accrual was suspended and trial was terminated based on the results of a pre-planned interim futility analysis. In terms of safety, treatment-related adverse events ≥ grade 3 were substantially higher for the investigational arm (45%) *versus* control (9%).^8^

## EXPERT OPINION

Unfortunately, we now have two failed clinical trials for PEGPH20. It is of both academic and clinical importance to consider possible explanations for these failed trials. In SWOG 1313, the answer may lie more within the toxicity of the entire regimen. As a consequence, the overall treatment exposure in the experimental arm was inferior. The study group received half the number of cycles of chemotherapy and more dose reductions than the control group. This substantial imbalance in treatment exposure is likely the most relevant factor contributing to the very poor outcomes of the study group. Dosing of PEGPH20 could be another factor that contributed to the negative results of SWOG 1313. The recommended phase II dose (RP2D) schedule for PEGPH20 was q2 weeks, whereas in the positive HALO-202 trial it was administered weekly. Additionally, the lack of subject selection based on the HA high biomarker likely played a role.

However, this was rectified in the HALO 301 study, which only included patients that had tumors with high-levels of HA expression. The phase II study showed promising results, especially in this subset of patients. The R2PD with the twice weekly dosing for PEGPH20 was also applied on this trial. Patients were also given enoxaparin to avoid the venous thromboembolism risk. Despite these corrections, HALO 301 now failed. Halozyme announced that although response rate was similar to the control arm, no significant difference was seen. Could it be perhaps that our theory of targeting the desmoplastic response is simply not enough?

Perhaps it is not solely the desmoplastic reaction that is the cause of chemoresistance of pancreatic cells, but additional intrinsic factors at play. The pancreatic cancer microenvironment is also filled with immunosuppressive cell types, such as myeloid derived suppressor cells (MDSCs) and regulatory T-cells (Tregs), which in turn alter the effector function of cytotoxic T-cells, leading to evasion of the immune system. It has also been shown that the amount of Tregs in the pancreatic microenvironment corresponds to the Tregs found peripherally, which could result in systemic chemotherapy resistance.^14^ The location of the cytotoxic T-cells also is problematic; they are located at the front of pancreatic cancer and not so much in the center where they can target malignant cells. Also, when they do cluster next to malignant cells they are unable to act upon them due to the dense stromal tissue.^15^ This has explained the poor response to immunotherapies in pancreatic cancer as well, along with the low mutational burden, and lack of significant neoantigens. Although preclinical studies have shown promise in the area of suppressing Tregs, clinical studies have not followed suit. The ECLIPSE trial, which depleted Tregs with cyclophosphamide and the GVAX vaccine has not shown any significant clinical outcomes.^16^

The epithelial to mesenchymal transition (EMT) is the conversion of epithelial cells into mesenchymal like cells in cell culture conditions, which is seen in the pancreatic tumor cell.^20^ However, the inhibition of this has been not proven to have an effect on pancreatic metastasis. Paradoxically, it does seem to affect chemoresistance of the pancreatic tumor cell. The theory behind this is the cell has seemed to have compensatory mechanisms to overcome the inhibition in terms of metastasis, but may suppress drug transporter and concentrating proteins, which could affect resistance to chemotherapies such as gemcitabine. Therefore, perhaps adding epithelial–mesenchymal transition (EMT) inhibitors to standard backbone chemotherapies is necessary to consider.

It is also important to consider that although the tumor stroma may be a physical barrier hampering drug delivery, it may also have protective effects in restraining tumor growth and progression. Sonic hedge hog (SHH) deficient tumors have reduced stromal content but surprisingly such tumors were more aggressive, exhibited undifferentiated histology, increased vascularity, and heightened proliferation.^21^ Another study investigating role of adjuvant gemcitabine compared to observation in resectable pancreatic cancer patients analyzed tissue samples of 162 patients. They found that dense stromal reaction was actually associated with improved disease free survival (DFS) and OS with median DFS and OS of 13.8 and 28 months in weak stroma (*p*=0.05) *versus* 10.1 and 20.2 months in strong stroma (*p*=0.047).^22^ Therefore, reversal of hypovascular stroma with stromal depleting and penetrating agents such as PEGPH20 may simply unleash the strain of aggressive cancer clones potentiating their metastatic capacity.

In summary, the phylogenesis of pancreatic cancer is much more complex than originally thought. It may indeed be a combination of stromal modifying agents as well as other strategies to overcome chemoresistance to better fight pancreatic cancer. Further studies in molecular biology to better characterize the complex interaction between the microenvironment and cancer cells are warranted.

## Figures and Tables

**Table 1.** Key Trials in Treatment of Pancreatic Cancer

Trial (year)	Number of Patients	Disease	Treatment	Median Survival (months)	*p*-value
**Chemotherapy**					
Burris et al^17^	126	Metastatic	Gemcitabine Fluorouracil	5.6 4.4	0.0025
Conroy et al^3^	342	Metastatic	FOFLIRINOX Gemcitabine	11.1 6.8	<0.001
Von Hoff et al^4^	861	Metastatic	Gemcitabine Gemcitabine+ nab-paclitaxel	8.5 6.7	<0.001
Ueno et al^18^	834	Locally advanced or metastatic	S1 alone Gemcitabine alone	9.7 8.8	<0.001 for non-inferiority
**Targeted Therapy**					
Moore et al^5^	569	Locally advanced unresectable or metastatic	Gemcitabine+Erlotinib Gemcitabine+Placebo	6.2 5.9	0.038
Golan et al^6^	154	Metastatic with germline *BRCA* mutation	Maintenance Olaparib Placebo	18.9 18.1	0.68
**Immunotherapy**					
O’Reilly et al^19^	65	Metastatic	Durvalumab+ Tremelimumab Duravlumab	3.1 3.6	Not Available
Le et al^16^	213	Metastatic	Cy/GVAX+CRS207 Physician’s choice chemo	3.7 4.6	Not Significant

Cy=cyclophosphamide; FOLFIRINOX=5-fluorouracil/leucovorin, irinotecan, oxaliplatin;GVAX=Vaccine consisting of two human allogeneic pancreatic tumor cells lines irradiated to release antigen and transfected with DNA to release granulocyte-macrophage colony-stimulating factor, CRS207=double deleted Listeria Monocytegenes, engineered to secrete mesothelin into antigen presenting cells.
